# Oxidized cholesterol exacerbates toll-like receptor 4 expression and activity in the hearts of rats with myocardial infarction

**DOI:** 10.34172/jcvtr.2020.07

**Published:** 2020-01-30

**Authors:** Arash Khorrami, Mojtaba Ziaee, Maryam Rameshrad, Ailar Nakhlband, Nasrin Maleki-Dizaji, Alireza Garjani

**Affiliations:** ^1^Medicinal Plants Research Center, Maragheh University of Medical Sciences, Maragheh, Iran; ^2^Department of Pharmacology, Faculty of Pharmacy, Tabriz University of Medical Sciences, Tabriz, Iran; ^3^Cardiovascular Research Center, Tabriz University of Medical Sciences, Tabriz, Iran; ^4^Natural Products and Medicinal Plants Research Center, North Khorasan University of Medical Sciences, Bojnurd, Iran; ^5^Research Center for Pharmaceutical Nanotechnology, Tabriz University of Medical Sciences, Tabriz, Iran

**Keywords:** Oxidized Cholesterol, Toll-Like Receptor 4, Inflammation, Myocardial Infarction, Cytokine, Rats

## Abstract

***Introduction:*** The present study examined the effects of high cholesterol and high oxidized-cholesterol diets on the myocardial expression of TLR4 and pro-inflammatory cytokine in rats.

*** Methods:*** Male Wistar rats were allocated into 6 groups and fed with a normal diet, cholesterol, and oxidized-cholesterol rich diets with or without isoproterenol-induced myocardial infarction. TLR4 and MyD 88 expression and levels tumor necrosis factor alpha (TNF-α) and interleukin 6 (IL-6) were measured in the heart and serum.

*** Results:*** Oxidized cholesterol-fed animals had higher serum levels of oxidized low-density lipoprotein (LDL) (263 ± 13 ng/dL) than the cholesterol-fed animals (98 ± 8 ng/dL; *P* < 0.001). A high level of oxidized-LDL caused fibrotic cell formation and enhanced neutrophil infiltration in the absence of MI. Both cholesterol and oxidized-cholesterol upregulated TLR4 mRNA expression and increased TNF-α and IL-6 production in the hearts of rats with MI. In rats fed with oxidized-cholesterol the serum and myocardial levels of TNF-α (653 ± 42 pg/mL, 1375 ± 121 pg/100 mg, respectively) were higher than MI group (358±24 pg/mL, *P* < 0.001 and 885 ± 56 pg/100 mg, *P* < 0.01). A significant correlation was seen between TLR4 expression and infarct size.

*** Conclusion:*** These findings suggest that cardiac TLR4 is preferentially upregulated by oxidized cholesterol in rats. Oxidized cholesterol may have a critical role in cardiac toxicity in the absence of pathological conditions.

## Introduction

Hypercholesterolemia is a major cardiovascular risk factor and is distinguished by a high level of low-density lipoprotein (LDL) and total cholesterol (TC) in patients’ blood.^1^

Oxidative modification of the lipids has been of interest because of their role in human health. Like other lipids, cholesterol-containing foods such as eggs, meats, and butter are susceptible to oxidation by cooking or processing and even prolonged storage in the presence of oxygen. The western dietary pattern contains 5%-10% oxidized cholesterol.^2^ In a plethora of studies have demonstrated that oxidized cholesterol products involved in many undesirable processes such as inhibition of cholesterol biosynthesis, signaling mechanisms, cytotoxicity and changes in membrane morphology and function and presumed risk factor for atherosclerosis and related cardiovascular dysfunction.^3,4^ Oxidized cholesterol can be absorbed from the gut into the bloodstream and incorporated to LDL and chylomicrons.^5^ Several studies have also provided substantial shreds of evidence that support the hypothesis that oxidized lipoproteins, especially oxidized LDL, play a pathogenic role in cardiovascular diseases (CVDs).^6^ Oxidized LDL is a potent inducer of inflammatory molecules and can produce inflammation in arteries, thus promotes atherosclerosis and enhances the risk of heart attack or stroke. Toll-like receptors (TLRs) are innate immune system components that detect pathogenic molecular patterns such as lipopolysaccharide in sepsis and develop the inflammatory response including expression of cytokines. TLR 2, 3, 4 and 6 are identified in heart myocytes amongst TLR family members.^7^ However, until yet, the best characterized TLR in CVDs is TLR4.^8^ TLR triggers a variety of pathways of signaling, including the primary and essential adaptor protein, MyD88 (myeloid differentiation primary response protein). MyD88 activation promotes the translocation of the NFκB into the nucleus and causes the gene transcription of proinflammatory cytokines, notably tumor necrosis factoralpha (TNF-α) and interleukin 6 (IL-6).^9,10^

Induction of cardiac dysfunction and failure after myocardial infarction is of great complexities. The excessive peripheral sympathomimetic discharge, oxidative stress, and inflammatory reactions contribute to the disease progression.^11,12^ Isoproterenol is a synthetic agonist of β-adrenoceptor which discharges the energy sources of cardiac cells and promotes the production of highly cytotoxic free radical and lipid peroxidation.^13^ therefore, induces myocardial infarction and leads to irreversible damage to cells and eventually infarct-like necrosis.^14^

In the present study, the myocardial infarction model is induced by isoproterenol administration to assess the impact of oxidized and non-oxidized cholesterol-enriched diets on normal and infarcted cardiac tissues. Furthermore, to compare the effect of a high level of LDL and oxidized LDL on inflammatory pathways following myocardial infarction we also assessed TLR4 expression and activity in the heart.

## Materials and Methods

### Experimental protocol

Thirty-six male Wistar rats (250 ± 20 g) were obtained from Razi Institute Karaj, Iran. The animals were housed under normal conditions (20°C-25°C, 70%-80% humidity, 12-hour light/dark cycle) for 2 weeks and then randomly assigned into 6 groups (n = 6). They received standard diet and water *ad libitum*.

The standard diet (percentage of each component) contained: 61.5% carbohydrate, 19.5% protein; 3.5% total lipid (0.05% cholesterol), 4.5% crude fibre; 2%mineral salt and 9% moisture. The high cholesterol (2% w/w) diet was prepared fresh every 3 days by supplementing 2 g powdered solid cholesterol (Sigma, Germany) into 98 g ground standard diet. The composition was kneaded into a dough by adding distilled water, pelleted and dried in an oven (50°C) during the night. The prepared meals were stored in dark and airtight containers in the refrigerator for no more than 3 days before use because of the prevention of the oxidative modification of cholesterol.^15^ The oxidized cholesterol-rich diet was prepared using the method described earlier by Staprans et al.^16^ After adaptation period, animals were allocated into six groups as follows: Group I—received standard lab diet+ isotonic saline, group II—high cholesterol-enriched diet+ isotonic saline, group III—received oxidized cholesterol-enriched diet+ isotonic saline, group IV—received standard diet + Isoproterenol (Iso) injection, group V—high cholesterol-enriched diet + Isoproterenol (Iso) injection, group VI—received oxidized cholesterol-enriched diet + Isoproterenol (Iso) injection.

All animals received mentioned diets for 14 weeks. At the end of the feeding period, myocardial infarction (MI) was induced by subcutaneous (SC) injection of isoproterenol (ISO, 100 mg/kg) at an interval of 24 hours for 2 consecutive days on the back part of animals^17^. The control rats in each diet group were given isotonic saline (0.5 mL, SC) instead of isoproterenol. On the third day after induction of MI, the animals were anesthetized and vein blood was collected for biochemical analysis. Subsequently, the heart was excised and stored deep-frozen in liquid nitrogen after washing with ice-cold normal saline.

### Serum biochemical analysis

Serum levels of TC, triglycerides (TG), LDL and high-density lipoprotein (HDL) were measured to evaluate the effect of fat-rich diet on lipid profile of experimental animals by available commercial kits (Pars Azmoon Laboratories, Iran). The assays were performed in duplicate according to the instructions of the manufacturer. Oxidized LDL (OxLDL) level was measured using OxLDL kits purchased from Glory Company. Enzyme-linked immunosorbent assay (ELISA) kits work upon a double-antibody sandwich method to determine the level of OxLDL in the sera.

### Histopathological examination

For the histopathological examination, the cardiac apex was cleaved and set in a neutral buffered formaldehyde solution. For cardiac myofibril and interstitial tissue assessment, the tissues were embedded in paraffin, sectioned at 5 μm and stained with Gomeri trichrome. In each segment of the cardiac apex, the severity of myocardial fibrosis was evaluated using a morphometric point-counting technique. Histopathological alterations ranked 1, 2, 3, and 4 respectively for low, moderate, high, and intensive pathological changes.

### Measurement of necrotic tissue in the heart

In another set of experiments, hearts (n = 6) from each group were harvested and subjected for measurement of infarct size (necrotic tissue). To determine the infarct size, animals were sacrificed and their hearts were removed, weighed, frosted and then cut into 2 mm width sections parallel to the atrioventricular groove. Tissue slices were embedded at a 1% (w/v) 2,3,5-triphenyl tetrazolium chloride solution in phosphate buffer, (pH 7.8) at 37°C for 20 minutes. In this method, viable cells colored red while the necrotic tissue (infarct area) remained unstained. Tissue slices were traced on acetate layers. After fixing the slices for an overnight in 10% (v/v) formalin, a digital computed photodetection planimetry (Image Pro Plus, version 4.5) was conducted on histologic slides to quantify the necrotic cells. The scale of the infarcted area was demonstrated as the percentage of left ventricular section.^18^

### Western blot analysis

Western blot analysis has been carried out according to the technique described earlier by Omar et al.^19^ Following the excision of the hearts from each group (n = 6), left ventricular tissues were homogenized in ice-cold solution containing 50 mM Tris–HCl, 150 mM NaCl, 5 mM Sodium Pyrophosphate (NaPPi), 50 mM NaF, 1 mM EDTA, 1 mM dithiothreitol (DTT), 0.1%SDS (w/v), 1% TXT-100 (v/v), and protease inhibitor cocktail (Roche, Mannheim, Germany). Consequently, the homogeneous tissues centrifuged at 4°C at 10 000 rpm for 10 minutes. For further analysis, the supernatant is aliquoted and stored at −80°C. The protein content of the samples was measured using the Bradford Protein Assay kit. The sample loading buffer was added to samples and 50 μg of the homogenous protein was exposed to SDS-Polyacrylamide gel electrophoresis by a Bio-Rad mini protean tetra system (Hercules, CA). The isolated proteins on gel have been loaded onto an Immobilon-P membrane (Millipore, Billerica, MA) and blocked in 5% fat-free milk in Tris-buffered saline-Tween-20. The membranes were subsequently tested using primary antibodies raised against MyD88 and GAPDH (1:1000 dilution). The membranes were eluted three times and incubated with peroxidase-conjugated goat anti-rabbit and rabbit anti-mouse secondary antibodies (1:5000 dilution). Following the extensive wash of the membranes, the BM Chemiluminescence kit (Roche, Mannheim, Germany) was used to demonstrate the antibodies. Image J software (Wayne Rasband, National Institute of Health, USA) was used to evaluate the densities of the immunoblots. MyD88 densitometric values have been standardized to GAPDH.

### Detection of the TLR-4mRNA in cardiac tissue by quantitative real-time PCR

In compliance with the manufacturer’s instructions, complete RNA from frozen left ventricular was obtained using TRI reagent (Sigma, St. Louis, USA). Agarose electrophoresis was applied to evaluate the integrity of extracted RNA while the purity of RNA was analyzed by optical density tests (A260/A280 Ratio) with Nanodrop instrument (ND 1000, Wilmington, USA). cDNA synthesized using 1 μg of each extracted RNA sample. Random Hexamer Primer and RevertAidTM H Minus M-MuLV Reverse Transcriptase (Fermentas, St. Leon-Rot, Germany) were used to conduct real-time PCRs. The processes were completed with the iQ5 optical system (Bio-Rad Laboratories Inc., Hercules, CA) in a total volume of 25 μL. This 25μL reaction mixture includes 1 μL of cDNA, 1 μL of primary (100nM of each primary) and 12.5 μL 2X SYBR Green PCR Master Mix (Applied Biosystems, Foster City, USA) and 10.5 μL of RNase/DNase Free water. All reactions were replicated three times and each experiment included negative control as well as NTC. The conditions of cycling were: 1 cycle in 10 minutes at 94°C, 95°C in 15 seconds, 53°C (annealing temperature) in 30 seconds and 72°C in 25 seconds. The target gene was standardized to the 18S internal reference gene for quantification. The manufactured primers are typically designed for the detection of the expressions of the TLR4 gene as described below:

For TLR4,

forward: 5′-AAGTTATTGTGGTGGTGTCTAG-3′;

reverse: 5′-GAGGTAGGTGTTTCTGCTAAG-3′.

For 18S rRNA,

forward: 5′-ACACGGACAGGATTGACAGATTG-3′;

reverse: 5′- GCCAGAGTCTCGTTCGTTATCG-3′.

Interpretation of the result was performed using the Pfaffle method.^20^

### Serum and cardiac assessment of TNF-α and IL-6

TNF-α and IL-6 were assessed with an ELISA in the serum samples and cardiac tissue (Rat TNF-α and IL-6 kits, IBL, Hamburg, Germany) based on the manufacturer’s instruction. Briefly, homogenized or mixed cardiac tissues have been kept with the ice-cold comprising 50 mM Tris–HCl, 150 mM NaCl, 5 mM Sodium Pyrophosphate (NaPPi), 50 mM NaF, 1 mM EDTA, 1 mM dithiothreitol (DTT), 0.1%SDS (w/v), 1% TXT-100 (v/v), and protease inhibitor cocktail (Roche, Mannheim, Germany). At 10 000 (rpm) for 10 minutes at 4°C, all samples were twice centrifuged. For the assay, we used the obtained supernatants. The cytokines were shown as pg/100 mg of cardiac tissue or pg/mL of serum.

### Neutrophil counting in blood

The number of neutrophils in blood samples was determined by the microscopic method. Pre euthanized Blood samples were drained before the authorization of animals and prepared with the Giemsa staining method. Finally, neutrophils were calculated at 100x zooming.

### Myeloperoxidase assay

Myocardial infiltration of neutrophil was evaluated using myeloperoxidase (MPO) measurement. In a solution consisting of 0.5% hexadecyl trimethyl-ammonium bromide (HTAB) dissolved in 50 mM potassium phosphate buffer (pH 6), cardiac tissues were homogenized to solve and extract the enzyme. At 4500 rpm for 20 minutes in the 4°C, the samples were centrifuged. The samples have been frozen and defrosted three times, after which sonication was repeated for 20 seconds. An aliquot of the supernatant (0.1 mL) or standard (Sigma, Germany) was then allowed to react with 2.9 mL solution of 50 mM potassium phosphate buffer at pH 6 containing 0.167 mg/mL of O-dianisidine hydrochloride and 0.0005% H2O2. After 5 min, using 0.1 mL of 1.2 M HCl interrupted the reaction. A spectrophotometer at 460 nm was used to calculate the rate of absorption in solution (Cecil 9000, Cambridge, UK). The activity of MPO was shown in mU per gram weight of wet tissue.

### Statistics

The quantitative variables were presented as mean ± SEM. The statistical analysis was done using analysis of variance (ANOVA), followed by a Tukey HSD post hoc test to compare variables between different groups. *P* value <0.05 considered significant.

## Results

### Effects of dietary cholesterol and oxidized cholesterol on the lipid profile

Feeding rats with high cholesterol and oxidized cholesterol diets both significantly elevated the serum concentration of TC from 66.3 ± 4.5 mg/dL in normal rats to 126.2 ± 7 and 133.5 ± 7, respectively (*P* < 0.001). Similarly, serum levels of LDL and triglycerides were meaningfully increased (2–3 folds; *P* < 0.001) in both groups. As the level of LDL was 56.8 ± 4.3 and 67.4 ± 3.5 mg/dL in the cholesterol and oxidized cholesterol feed rats, whereas it was 21.2 ± 1.1 mg/dL in control animals. The serum level of oxidized cholesterol was significantly higher both in the cholesterol and oxidized cholesterol-fed animals. However, oxidized cholesterol-fed animals showed a very high level of oxidized LDL (263 ± 13 ng/dL) than the cholesterol-fed animals (98 ± 8 ng/dL; *P* < 0.001). The concentration of oxidized cholesterol in control rats was 34 ± 3.8 ng/dL.

### Histopathological examination of the cardiac tissues

**Figure 1:**
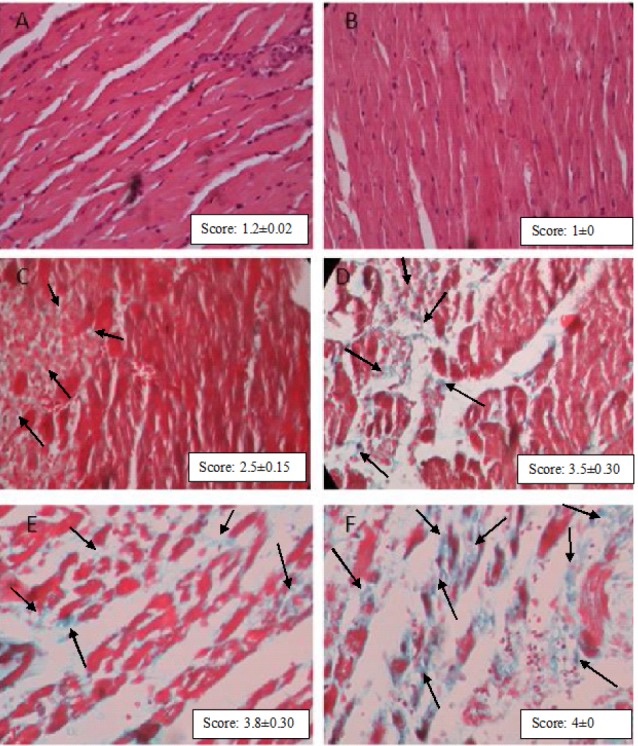
Representative microphotographs showing sections of rat cardiac apexes. Normal control group with clear striation myofibrils (1A); Cholesterol- rich diet group (1B); oxidized cholesterol group without MI shows considerable damage in myofibrils(1C, Blue Dots; indicated by arrows). In Iso-treated rats, intensive cardiomyocyte fibrosis was observed. (Dyed Blue; indicated by arrows) (1D). High levels of LDL and especially oxidized LDL in serum following myocardial infarction deteriorate the fibrosis (1E and 1F, respectively). Gomeri’s one-step Trichrome (40× magnification). Grading of histopathological changes in rat cardiac apex tissues was shown in the right corner of each photomicrograph. Data are expressed as mean ± SEM each group (n = 6).

The myocardial fibers were seen in a well-arranged manner with clear striations pattern without fibrosis or collapse in the myofibrils in the normal control group (Figure 1A) and the group feed with non-oxidized cholesterol (Figure 1B). However, feeding with oxidized cholesterol caused notable fibrotic damage to the heart tissues even if myocardial infarction was not present (Figure 1C). Histological characterization of the cardiac wall in isoproterenol-treated rats showed hypertrophia, extensive subendocardial necrosis, and excessive fibroblastic hyperplasia (Figure 1D). Feeding animals with high cholesterol and high oxidized cholesterol diet followed by isoproterenol administration activated the inflammatory response and exacerbate the hypertrophia and myocardial fibrosis from score 2.3 to 2.8 and 3.7 (*P* < 0.01) respectively, as shown in Figures 1E and 1F. Grading the histological sections damage showed that in addition to myocardial infarction, high oxidized cholesterol diet significantly (*P* < 0.05) increased the tissue damage score in non-infarct hearts (Figure 1C).

### Effects of cholesterol and oxidized cholesterol on infarct size

**Figure 2:**
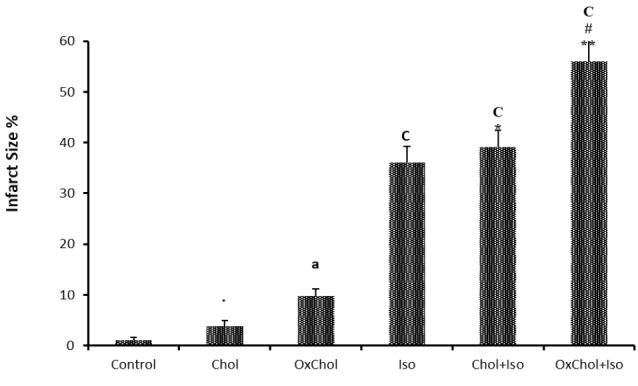
Myocardial infarction size of rats fed with normal (control), high cholesterol (Chol) and high oxidized cholesterol (OxChol) rich diets with or without Isoproterenol (ISO)-induced MI showed as the percentage of heart tissue. ^a^*P* < 0.01 vs. the control group; C *P* < 0.001 vs. the control group; * *P* < 0.05, **P < 0.01 vs. the ISO group; # *P* < 0.05 vs. the Chol+ISO group using one way ANOVA with Tukey post hoc test. Data are expressed as mean ± SEM each group (n = 6).

Feeding animals with diets containing cholesterol and oxidized cholesterol resulted in a significant increase in the size of myocardial infarction following ISO-induced myocardial infarction. As shown in Figure 2, the mean infarct size in heart of rats with ISO-induced MI which were fed with standard diet was 36.1 ± 3.1%, while feeding with oxidized cholesterol-enriched diets significantly increased the size of myocardial infarction up to 56.6 ± 5% (*P* < 0.001). Furthermore, infarct size in the oxidized Cholesterol-fed group was over 40% larger than the cholesterol-fed group (*P* < 0.01) among animals with MI. Also feeding animals with oxidized cholesterol also caused necrotic injury in heart tissue even without inducing MI.

### Effects of dietary cholesterol and oxidized cholesterol on MPO activity in the myocardium and neutrophil count in blood after myocardial infarction

Myocardial infarction induced with isoproterenol administration resulted in a significant (*P* < 0.001 vs control) increase in neutrophil infiltration into the myocardium, as evaluated by an increase in MPO activity in heart tissue (Table 1). Feeding animals with the oxidized cholesterol-enriched diet with myocardial infarction was found to increase the enzyme activity in the left ventricle from 3.4 ± 0.27 mU/g wet tissue in ISO group (MI) and 3.8 ± 0.35 in Chol+ ISO group rats to 4.65 ± 0.2 (*P* < 0.05). These results indicate that rats fed with oxidized cholesterol have a significant increase in neutrophil count (*P* < 0.05) compare to the control group. Also, rats with MI and fed with an oxidized cholesterol-enriched diet have higher neutrophil count compare to the ISO and Chol+ ISO groups (*P* < 0.05).

**Table 1:** The effect of dietary cholesterol and oxidized cholesterol on serum and myocardial IL6, TNF-α, myeloperoxidase (MPO) activity and Neutrophil count in the blood

	**Control**	**Chol**	**OxChol**	**Iso**	**Chol+Iso**	**OxChol+Iso**
Serum IL-6 (pg/mL)	42±3.5	51±4.4	85±7.1a	104±9^c^	127±11.9	216±16^***,###^
Myocardial IL-6 (pg/100 mg wet tissue)	37±2.9	39±3.3	53±5.3	89±7.5^c^	102±9	176±13^***,###^
Serum TNFα (pg/mL)	68±5	108±9	210±15^b^	358±24^c^	432±25	653±42^***,###^
Myocardial TNFα (pg/100 mg wet tissue)	250±22	319±27	588±46^a^	885±56^c^	1040±94	1375±121^**,#^
MPO activity (U/g)	2.245±0.1	2.23±0.2	2.9±0.1^5^	3.4±0.2^a^	3.5±0.2	4.65±0.2^**,#^
Neutrophil%	23±2	29±2	34±3^a^	40±3^a^	46±4	57±5^**,#^

^a^ *P* < 0.05,^b^*P* < 0.01, ^c^*P* < 0.001 from respective control value; *P < 0.05, **P < 0.01 and ***P < 0.001 compared with Isoproterenol (ISO) injected group and #*P* < 0.05, ##*P* < 0.01 and ###*P* < 0.001 compared to Chol+ ISO group using one way ANOVA with Tukey post hoc test. Data are expressed as mean ± SEM each group (n = 6).

### Effects of dietary cholesterol and oxidized cholesterol on the level of TNF-α in myocardium and serum after myocardial infarction

In the current study, myocardial and serum levels of TNF- α in rats fed with high-fat diets and isoproterenol-induced myocardial infarction animals were evaluated to clarify the correlation between TLR4/MyD88 protein expressions in myocardium and production of proinflammatory cytokines.

The results indicate that the level of TNF-α was considerably increased in animals with myocardial infarction compare to the control group. This increase is more than fivefold in serum and 3 fold in the myocardium (*P* < 0.001) (Table 1). It was found that feeding with cholesterol has a minimal effect on the TNF-α level in serum and myocardium in healthy animals. However, an oxidized cholesterol-enriched diet significantly elevated the TNF-α level in serum and myocardium (*P* < 0.01 and *P* < 0.05) respectively. In isoproterenol-induced myocardial infarction rats which fed with oxidized cholesterol-enriched diet TNF-α level in serum and myocardium (653 ± 42, 1375 ± 121) is considerably increased compared to ISO (358 ± 24, *P* < 0.001and 885 ± 56 *P* < 0.01) and Chol+ISO group (432 ± 25, *P* < 0.01 and 1040 ± 94, *P* < 0.05) (Table 1).

### Effects of dietary cholesterol and oxidized cholesterol on the level of IL-6 in myocardium and serum after myocardial infarction

To further clarification of the injurious effect of high-fat diets, the potential effect of dietary cholesterol and oxidized cholesterol on the level of IL-6, which is a major pro-inflammatory cytokine secreted following TLRs activation, was investigated in this study. The results showed that the level of IL-6 in myocardial tissue and serum samples increased more than 2 folds (*P* < 0.001). Feeding animals with an oxidized cholesterol-enriched diet in contrast to the cholesterol-enriched diet worsen the inflammatory condition in the myocardium. Serum and myocardium IL-6 level increased from 104 ± 9 pg/mL and 89± 7 pg/100 mg of wet tissue in the MI group to 216 ± 16 and 176 ± 13 in OxChol+ ISO group (*P* < 0.001) (Table 1).

### Effects of dietary cholesterol and oxidized cholesterol on TLR4 expression in the myocardium after myocardial infarction

**Figure 3:**
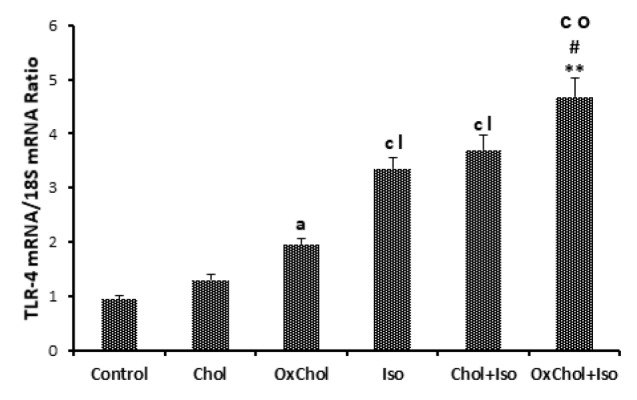
Effects of dietary cholesterol and oxidized cholesterol on TLR4 mRNA expression in myocardial tissues of rats following myocardial infarction. RNA was isolated and converted to cDNA. TLR4 mRNA expression was analyzed by PCR in real-time.
ISO, Isoproterenol; Chol, cholesterol; OxChol, oxidized cholesterol. ^a^*P* < 0.05, ^c^*P* < 0.001 from respective control value; ^l^*P* < 0.05, ^o^*P* < 0.001 as compared to OxChol. ^**^P < 0.01 as compared to Isoproterenol (ISO) injected group and ^#^*P* < 0.05 compared to Chol+ ISO using one way ANOVA with Tukey post hoc test. Data are expressed as mean ± SEM each group (n = 6).

In the present study, we showed that the TLR4 mRNA level in rats myocardium affected by isoproterenol increased up to 3.35 ± 0.2 which is 3 folds more than its value in normal animals (0.94 ± 0.08, *P* < 0.001). Feeding animals with a high cholesterol diet had no significant effect on the TLR4 mRNA level (Figure 3). However, the oxidized cholesterol-enriched diet increased this value to 4.67 ± 0.35 which is statistically significant compared to the ISO and Chol+ISO group (*P* < 0.01, *P* < 0.05) respectively.

### Effects of dietary cholesterol and oxidized cholesterol on MyD88 protein expression in the myocardium after myocardial infarction

**Figure 4:**
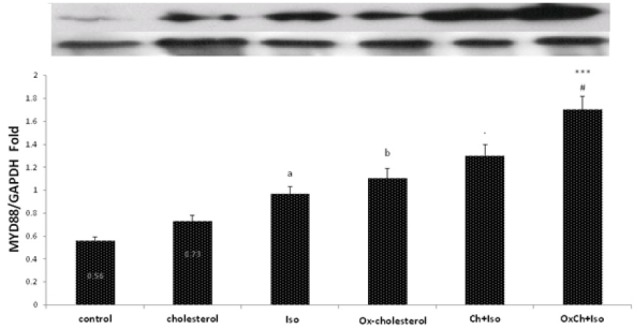
Representative immunoblots of MyD88 protein content following myocardial infarction with or without cholesterol or oxidized cholesterol-enriched diets were shown above the column. Bars represent the ratio of MyD88 to GAPDH. ISO: Isoproterenol; Chol: cholesterol; OxChol: oxidized cholesterol. ^a^*P* < 0.05, ^b^*P* < 0.01 from respective control value; *** *P* < 0.001 compare to the ISO group; ^#^ P<0.05 compared to Chol+ ISO using one way ANOVA with Tukey post hoc test. Data are expressed as mean ± SEM each group (n = 6).

TLRs are essential components of the innate immune response and stimulate NF-κB transcription factor via its intracellular adapter protein (MyD88). NF-κB transcription factor activation increases the systemic and tissue levels of TNFα and IL6.^9^ The specific purpose of the present study was to evaluate the effect of dietary fats on TLRs activity. Therefore, the MyD88 protein level in the left ventricle tissue was measured following high-fat diets feeding and MI induction by isoproterenol. We aimed to determine whether the high-fat diets could stimulate TLRs activity through MyD88 up-regulation or not. It was observed that dietary oxidized cholesterol contrary to nonoxidized cholesterol in healthy rat stimulates MYD88 protein production (*P* < 0.05). MYD88 protein content in infarcted myocardium reached to two times its value in the normal control group (*P* < 0.01). Dietary oxidized cholesterol significantly (*P* < 0.001) increased MYD88 protein content in infarcted myocardium in the same way that increased TLR4 expression compare to the ISO group (Figure 4).

### Toll-Like Receptor 4 expression correlation with infarct size

**Figure 5:**
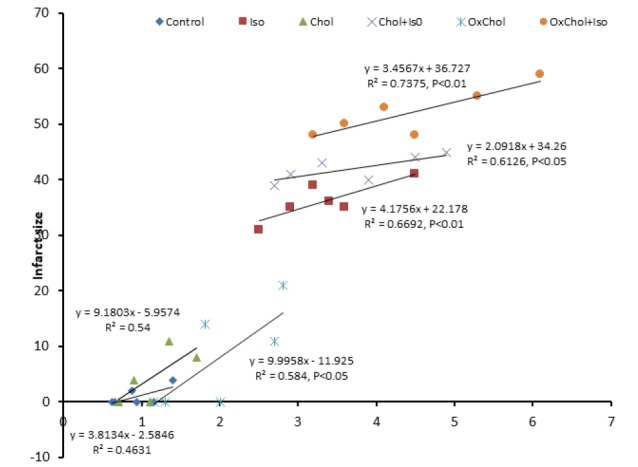
Correlation between TLR4 expression and infarct size in different groups.

A significant correlation was noted between TLR4 expression and infarct size (Figure 5) in OxChol group (r2 = 0.6) in healthy animals. In infarcted myocardium the significant correlation between TLR4 expression and infarct size in ISO (r2=0.67, *P* < 0.01), Chol+ISO (r2 = 0.62) and OxChol+ISO (r2=0.74, *P* < 0.01) was observed.

## Discussion

To date, the precise molecular mechanisms triggers for inflammation in atherosclerosis are not completely elucidated, but studies over past years have shown that human lipid-rich atherosclerotic lesions play a major causative role in immune and inflammatory responses.^21^

OxLDL appears to be a pivotal component in the ROS formation and the pathogenesis of atherosclerosis and secretion of cytokines and chemokines^22^ and inhibits 3-hydroxy-3-methylglutaryl coenzyme A reductase (HMG-CoA reductase) activity.^3^

TLRs play an important role in atherosclerosis as a chronic inflammatory disease. The variety of TLRs have been detected in cardiomyocytes. Activation of TLRs lead to elevation of cytokines such as TNF receptor-associated factor 6 (TRAF6), IL-1,6 receptor-associated kinases nuclear factor-kappaB (NF-κB) that play important roles in myocardial inflammation and infarction in both regional and global models and heart failure.^23^ Several experiments demonstrated inhibition of TLR4 has decreased myocardial damages following ischemia/ reperfusion injuries.^24^ TLR4 subsequently stimulates MyD88, leading to apoptosis and it could be a potential target for novel approaches to the treatment of CVD.^25^ We have demonstrated, in an isoproterenol-induced myocardial infarction model in rats, that TLR4 expression and MyD88 activation in the heart tissue is highly involved in myocardial dysfunctions.^9,10^ In the line with our results Chaves et al observed that TLR4 enhanced the immune response to OxLDL and increased T cells and this may be an important culprit in the pathogenesis of atherosclerosis.^26^

Interestingly, our results demonstrated that dietary OxLDL increased TLR4 and MYD88 protein production in infarcted heart tissue to two folds. Our findings support recent studies showing that OxLDL induces a proinflammatory response and elevated cytokine levels in response to TLR activation. OxLDL may promotes cytokines by TLR-signaling, the PI3K-AKT-pathway, enhance glycolysis, and rise histone H3K4 methylation.^27,28^ OxLDL could be classified as damage-associated molecular pattern (DAMP).^29,30^ DAMPs activate of smooth muscle cells and increased production of cytokines in response to restimulation.^29^ Other studies demonstrated that inhibition of TLR4/MyD88/NFκB signaling pathway could improve the severity of MI and prevent cardiac remodeling.^31^

We have recently reported that cardiac function in animals fed with high cholesterol and oxidized cholesterol diets before MI induction by isoproterenol considerably suppressed. In summary, our findings improve knowledge about the molecular mechanism of cardiac TLR4 receptors and the implication of dietary OxLDL in inflammatory and immune signaling. These results suggest that cardiac TLR4 is preferentially upregulated by oxidized cholesterol in rats. Oxidized cholesterol may participate as an important player in cardiac toxicity in the absence of pathological conditions.

OxLDL activated TLRs, critically influenced by cytokine and chemokine production in an innate immune response.

In summary, our results suggest that the high serum levels of OxLDL worsen post-infarction changes of the myocardium greater than non-oxidized LDL. Also, we demonstrate that OxLDL-induced TLR cascades that innate immunity mechanism. Further research is necessary to elucidate the detailed mechanisms of TLRs and the impact on the metabolic and inflammatory phenotypes. The pharmacologic intervention of these pathways might open a novel area to modulate atherosclerotic CVDs.

## Competing interests

None.

## Ethical approval

The study was approved by the Regional Ethics Committee of Tabriz University of Medical Sciences (Approval ID: TBZMED.REC.1394.721).

## Acknowledgments

Our investigation was supported by the grant from the research deputy of Tabriz University of Medical Sciences, Tabriz, Iran.
